# Supplementary material for: Characterization of the Survival Influential Genes in Carcinogenesis
Source: Int J Mol Sci. 2021 Apr 22;22(9):4384. doi: 10.3390/ijms22094384 (PMC8122717; doi:10.3390/ijms22094384)
Supplement: Supplementary file 1 [file ijms-22-04384-s001.zip › ijms-1184805-supplementary.pdf]

Supporting Information

Characterization of the survival influential genes in carcinogenesis

Divya Sahu, Yu-Lin Chang, Yin-Chen Lin, Chen-Ching Lin

Contents

Characterization of survival influential genes in carcinogenesis 1
Figures 2
Figure S1: Distribution of the identified SIGs across cancers. 2
Figure S2: Statistical power of harmful genes in each cancer type. 3
Figure S3: Statistical power of protective genes in each cancer type. 4
Figure S4: Comparison of SCC between genes forming PPIs. 5
Figure S5: Expression of clinically relevant genes in primary tumor and matched normal samples. 6
Figure S6 (part I): Survival estimates of overall survival in different cancer patients. 7
Figure S6 (part II): Survival estimates of overall survival in different cancer patients. 8
Figure S7: Differential expression of the survival influential genes. 8
Figure S8: Differential expression of the SIGs between censored and deceased samples. 9
Figure S9: Differential expression of the survival influential genes between censored early and late stage samples. 9
Figure S10: Overview of transcriptome expression in cancer genomes. 10
Tables 11
Table S1: Comparison of co-expressed degree among protective, harmful, and non-SIGs in cancers. 11
Table S2: Comparison of centrality between harmful SIGs and non-SIGs in static protein interaction network. 12
Table S3: Comparison of centrality between protective SIGs and non-SIGs in static protein interaction network. 13
Table S4: List of significant cancer relevant hallmarks in which the pan-cancer SIGs participate. 15
Table S5: Cancer type analyzed in the study and their abbreviations. 16
Datasets 17
Dataset S1: List of statistically significantly associated survival influential genes in each cancer. 17
Dataset S2: List of significant confounding risk factors identified from univariate Cox regression ($P < 0.05$) in different cancer types. 17

Figures

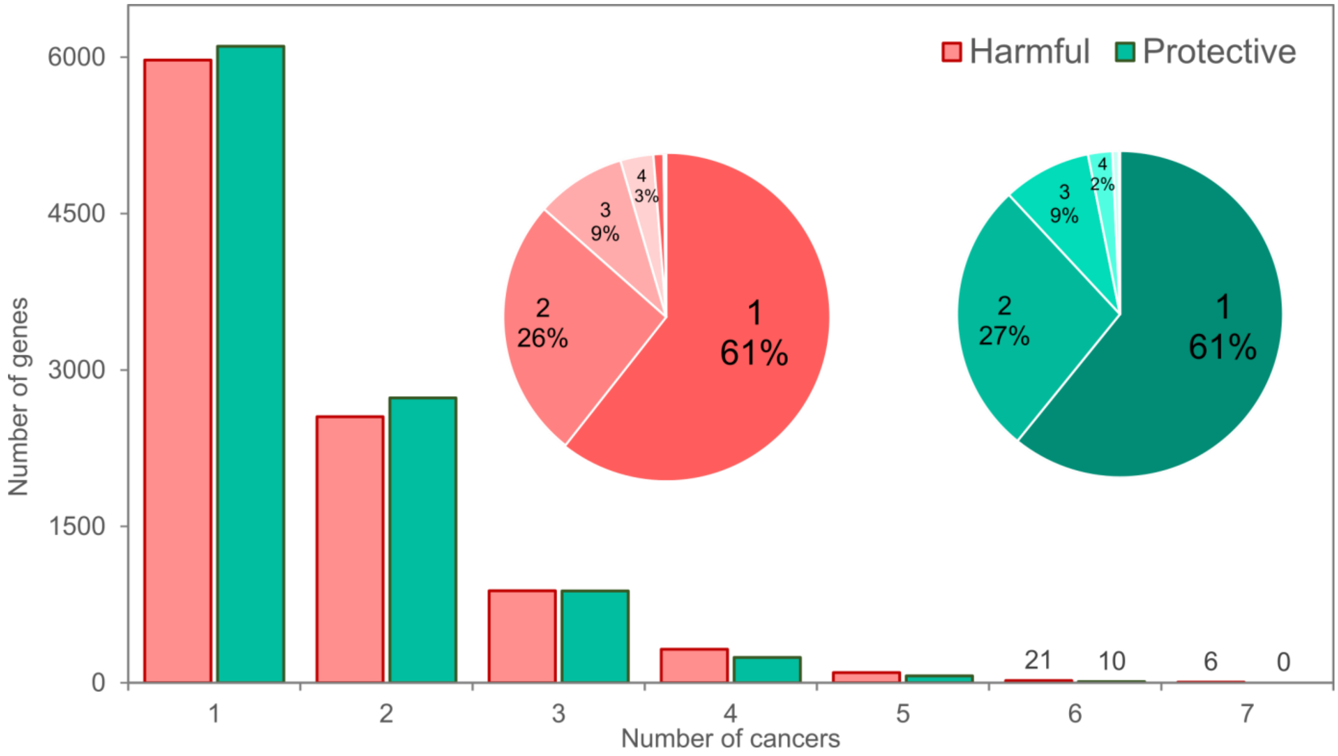

Figure S1: Distribution of the identified SIGs across cancers.

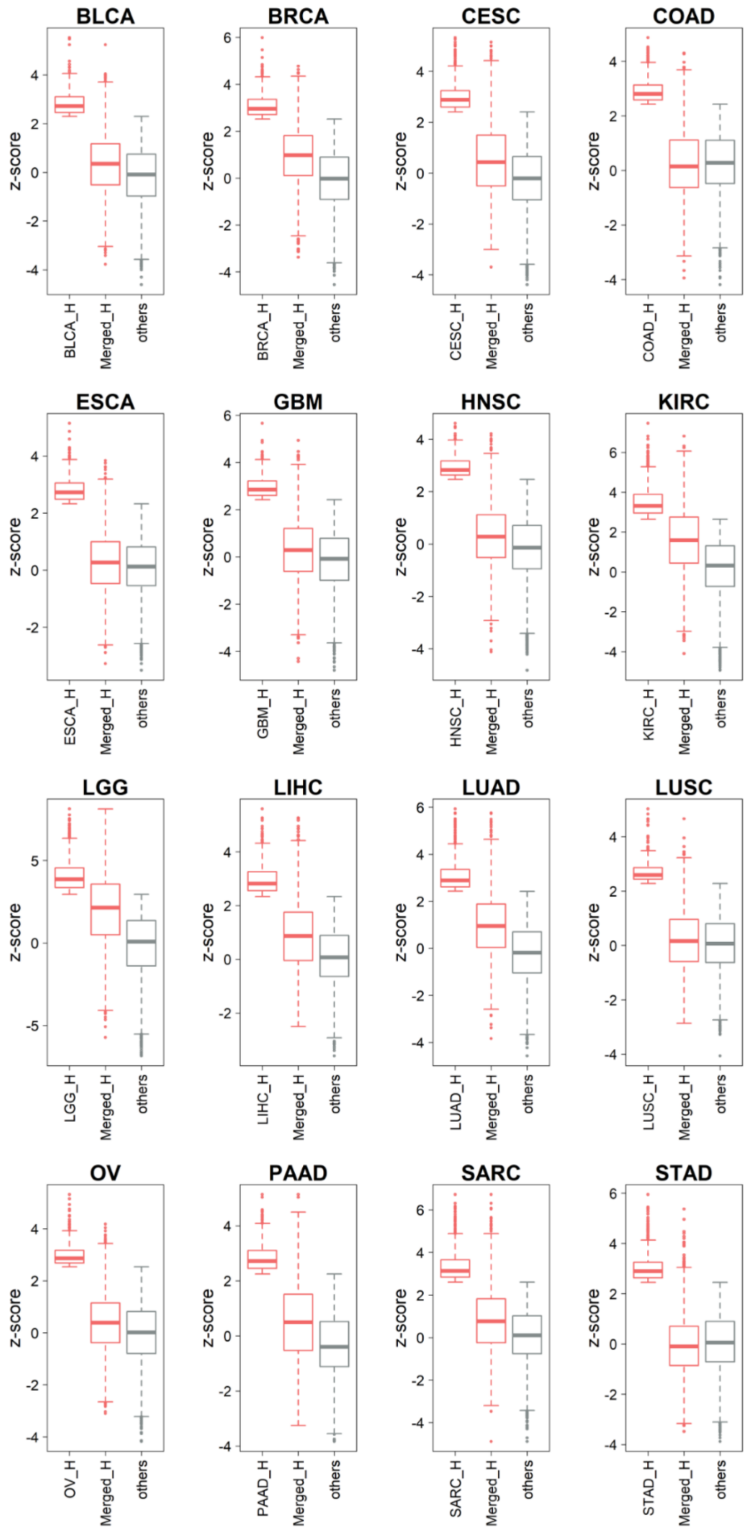

Figure S2: Statistical power of harmful genes in each cancer type.

Boxplot shows the distribution of z-scores of harmful genes in each cancer and merged cancer. Here others denote genes excluding harmful genes from each cancer and merged cancer.

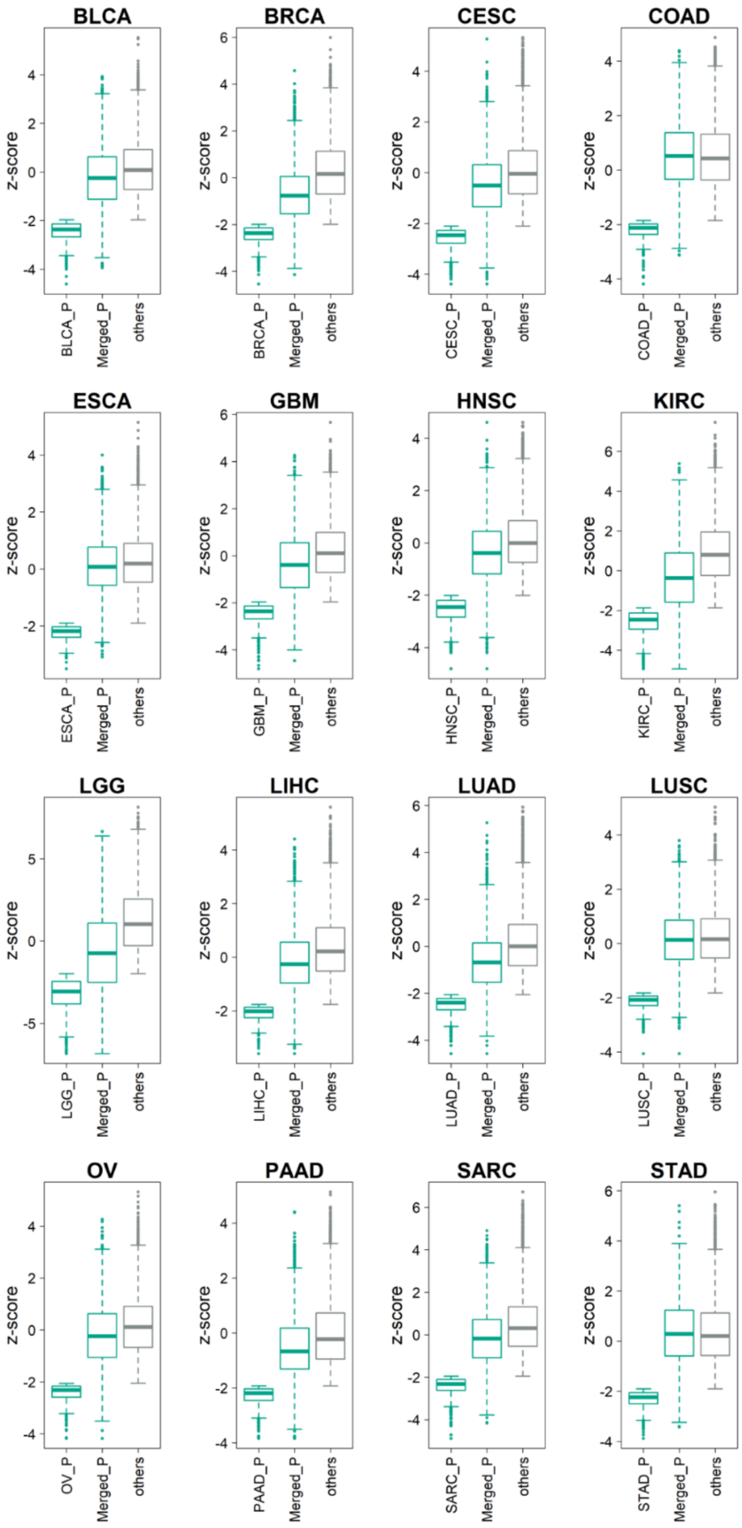

Figure S3: Statistical power of protective genes in each cancer type.

Boxplot shows the distribution of z-scores of protective genes in each cancer and merged cancer. Here others denote genes excluding protective genes from each cancer and merged cancer.

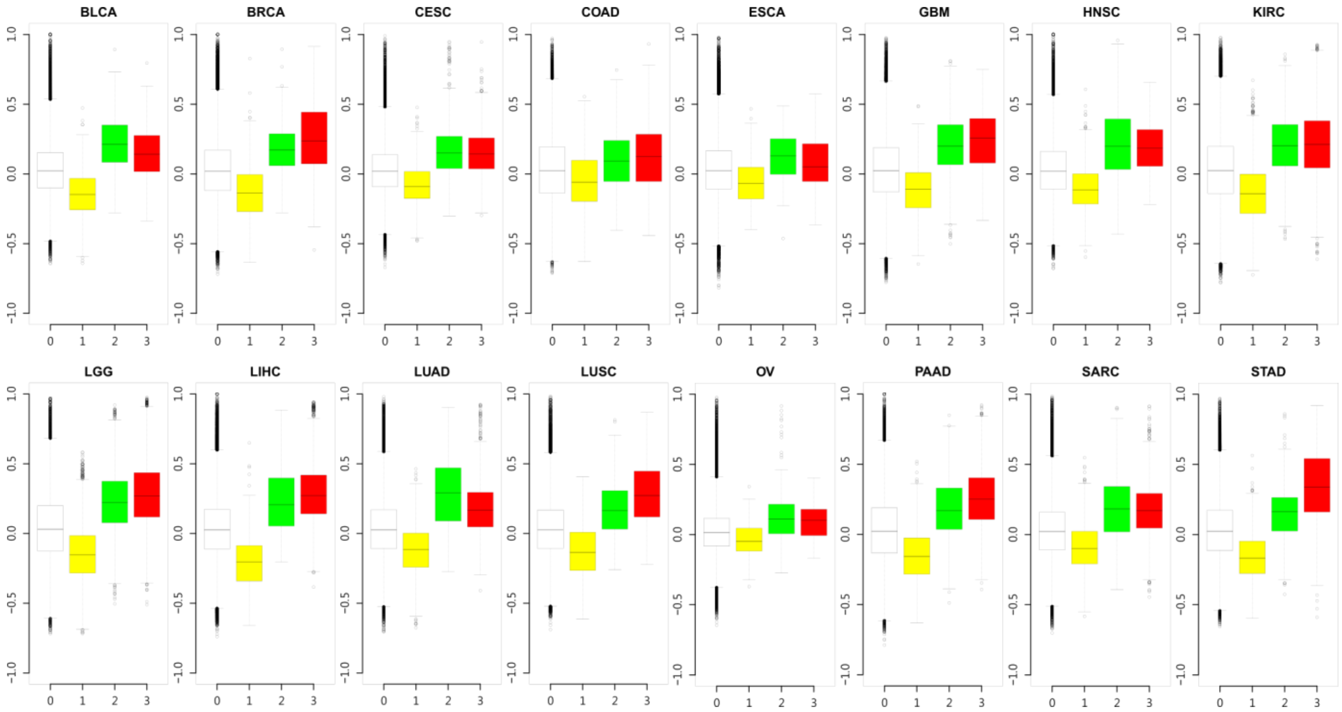

Figure S4: Comparison of SCC between genes forming PPIs.

The y-axis represents SCC between genes forming PPIs in different categories in the x-axis. The categories are 3: within harmful SIGs, 2: within protective SIGs, 1: between harmful and protective SIGs, and 0: between non-SIGs. Except for the comparisons between harmful and protective SIGs in CESC, COAD, ESCA, HNSC, KIRC, OV, and SARC, the remaining comparisons between two categories in each cancer are significant (p-value < 0.05, Wilcoxon rank sum test).

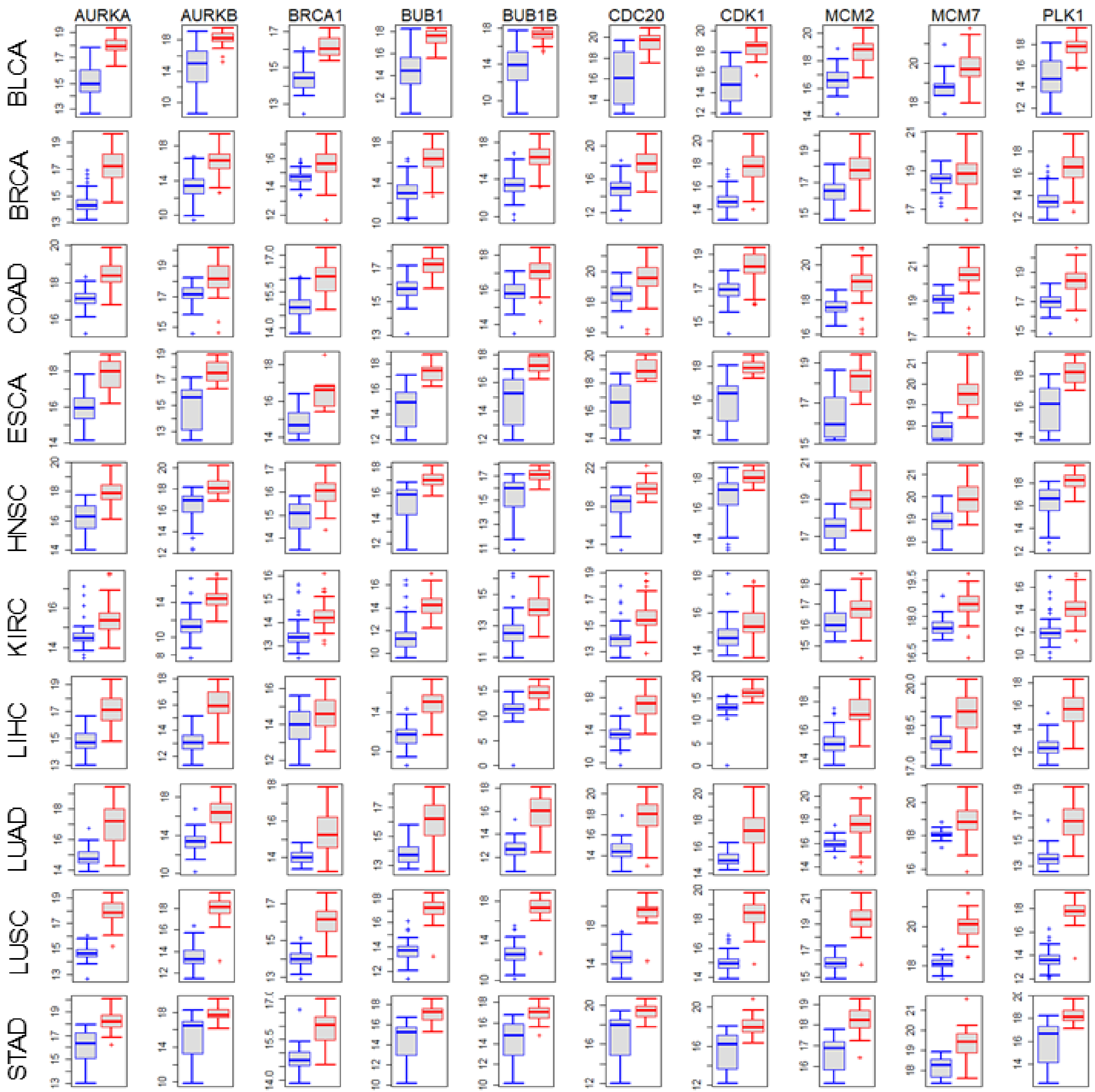

Figure S5: Expression of clinically relevant genes in primary tumor and matched normal samples.

The RSEM normalized expression value is displayed in the log2 (x+1) scale. The red boxes show the gene expression in primary tumor samples and blue boxes matched normal samples in each cancer type.

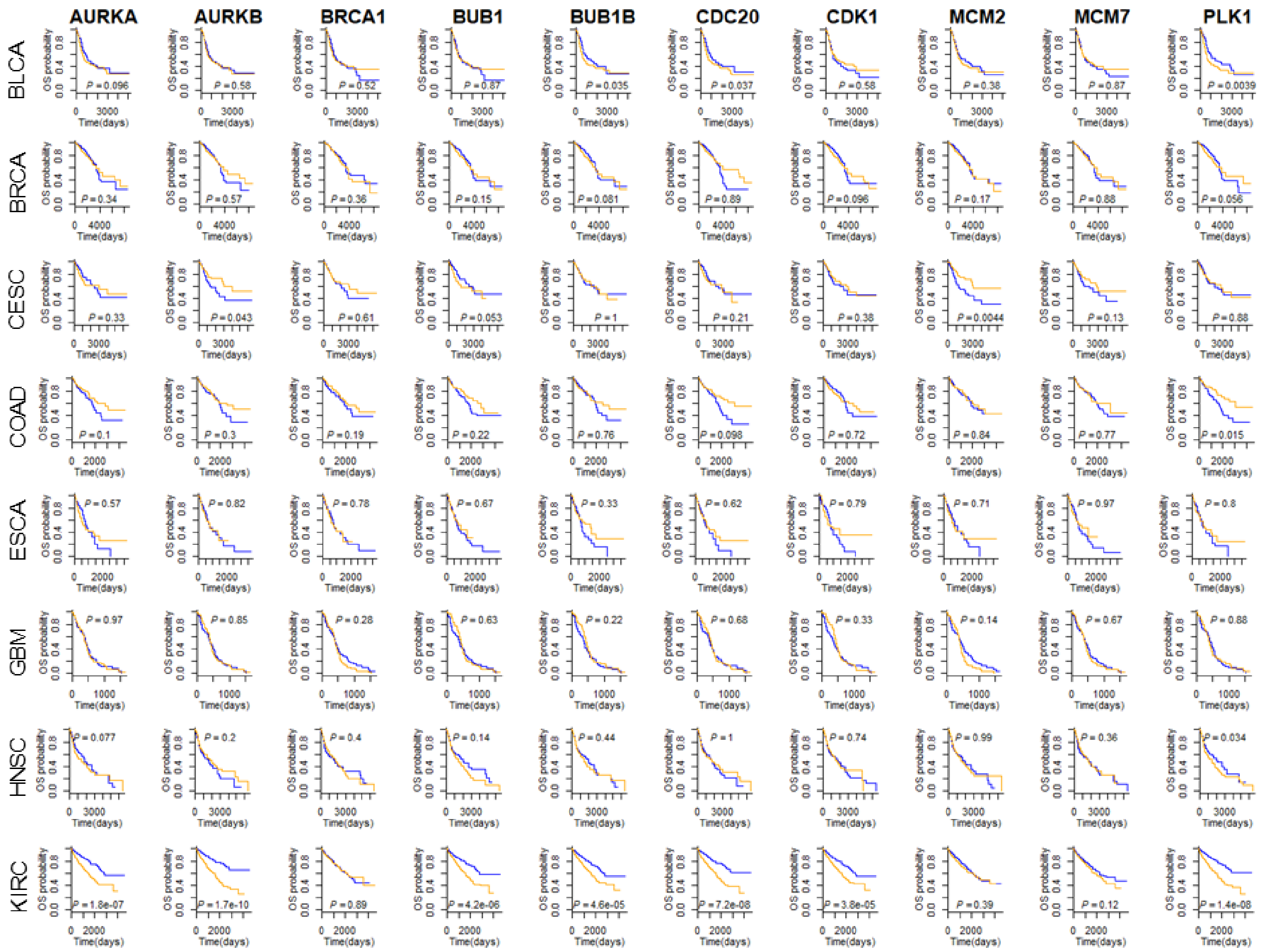

Figure S6 (part I): Survival estimates of overall survival in different cancer patients.

Kaplan-Meier plots of low expression and high expression groups based on the median expression. The *p*-values were obtained using a Mantel log-rank test.

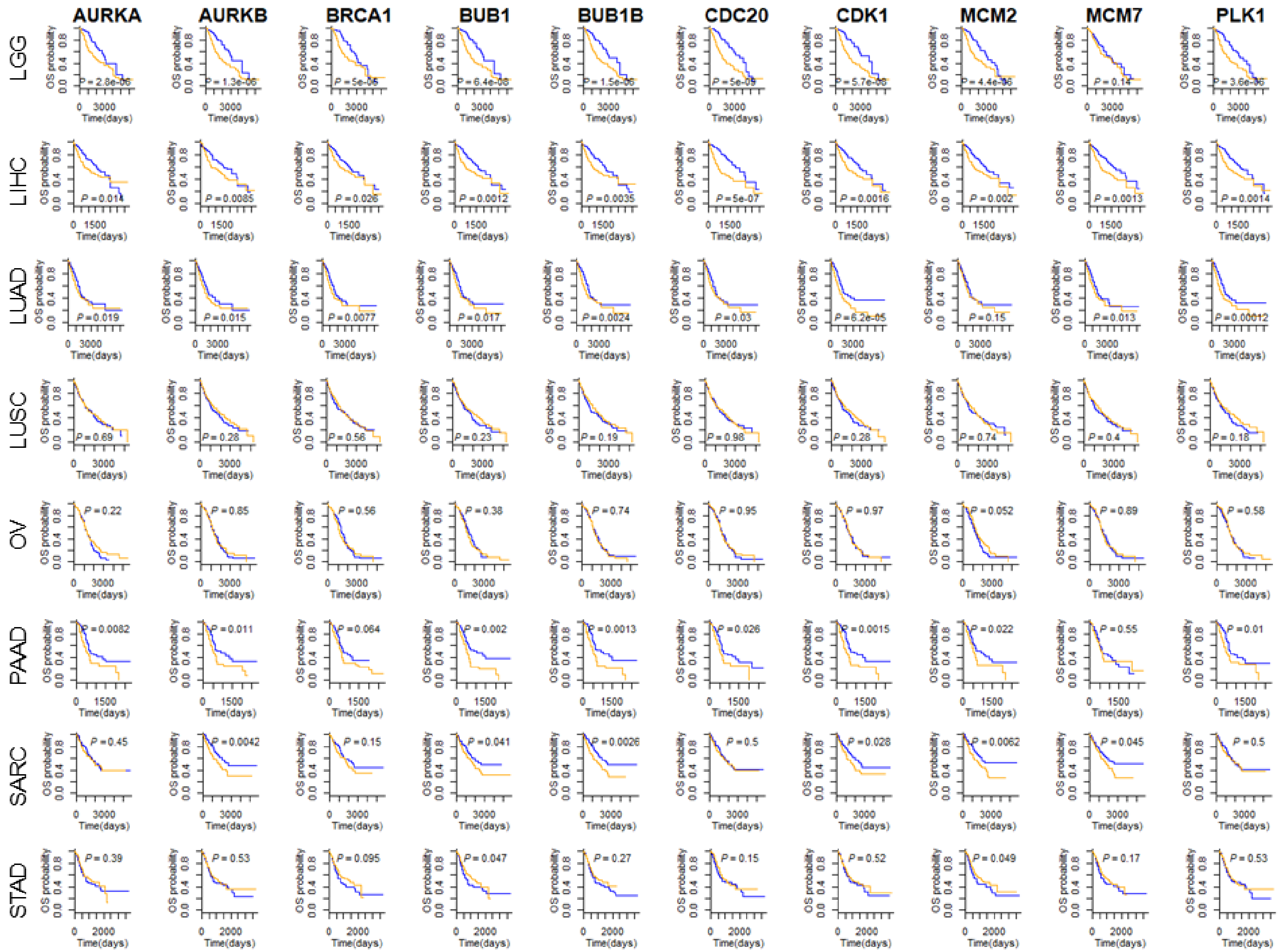

Figure S6 (part II): Survival estimates of overall survival in different cancer patients.

Kaplan-Meier plots of low expression and high expression groups based on the median expression. The *p*-values were obtained using a Mantel log-rank test.

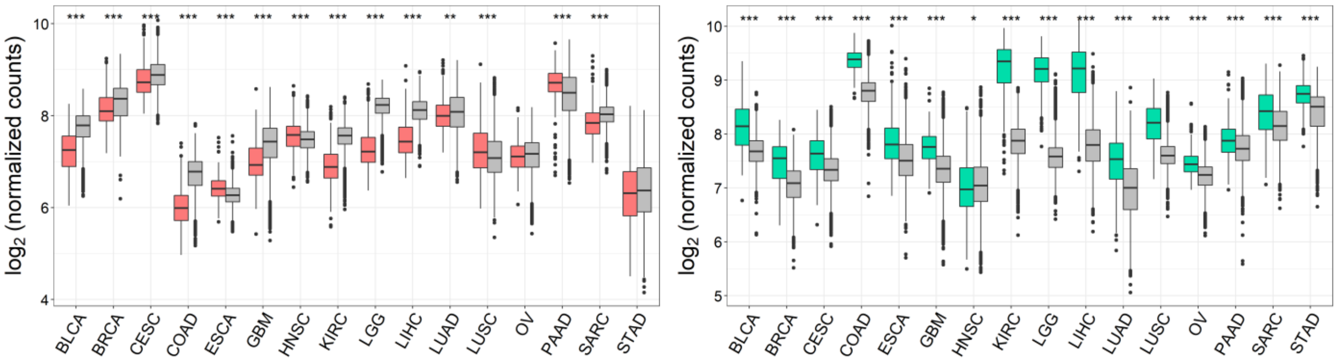

Figure S7: Differential expression of the survival influential genes.

Red (harmful) and green (protective) boxes show the expression level of the survival influential genes in the cancer type in which they are identified, labeled at x-axis. Grey boxes are expression level in 15 cancer types except for the cancer type at the x-axis. The *p*-values were obtained using the Mann-Whitney Wilcoxon test. * < 0.05, ** < 0.01, *** < 0.001.

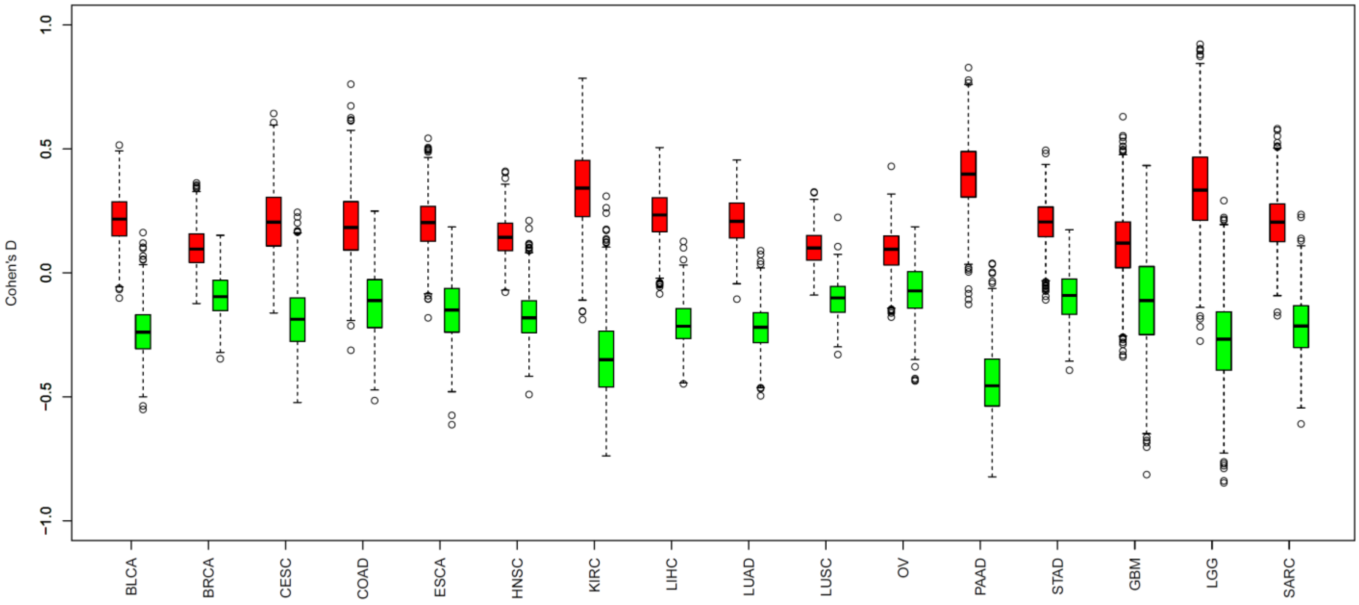

Figure S8: Differential expression of the SIGs between censored and deceased samples.

The expression difference of the SIGs between censored and deceased samples is measured by Cohen's D (deceased - censored). The red and green boxes represent harmful and protective SIGs, respectively.

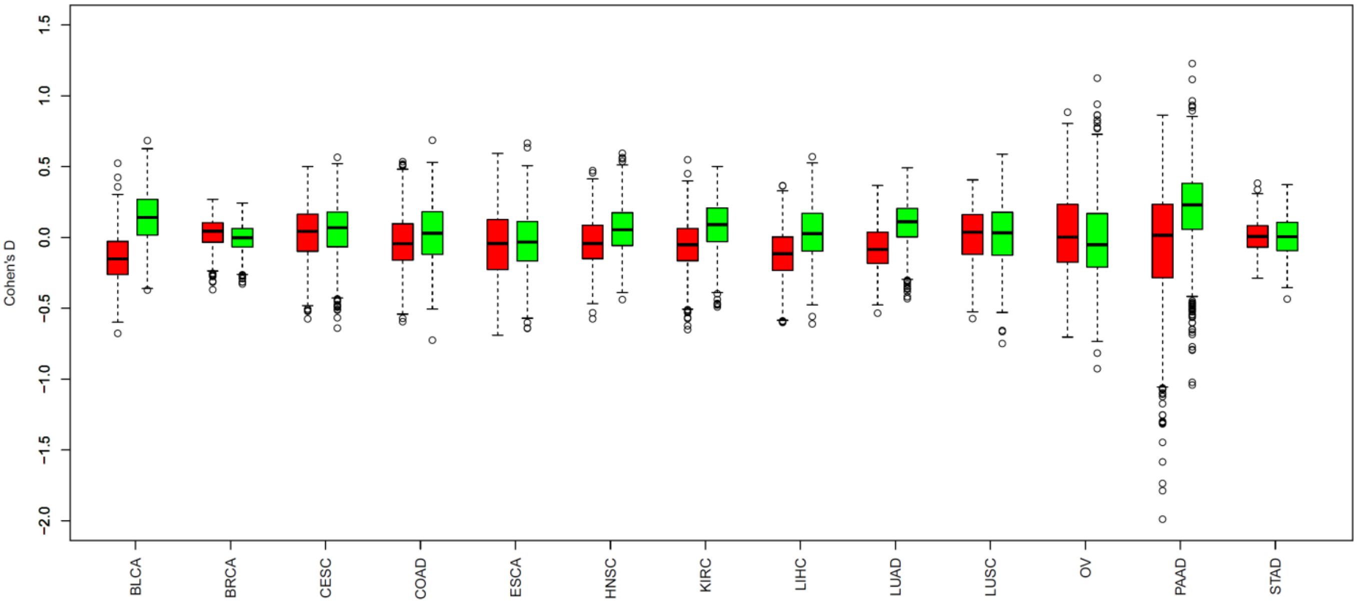

Figure S9: Differential expression of the survival influential genes between censored early and late stage samples.

The expression difference of the SIGs between censored samples with late and early stage tumors is measured by Cohen's D (early - late). Red boxes represent harmful genes and green boxes protective ones. Herein, GBM, LGG, and SARC are not shown due to lacking insufficient censored samples.

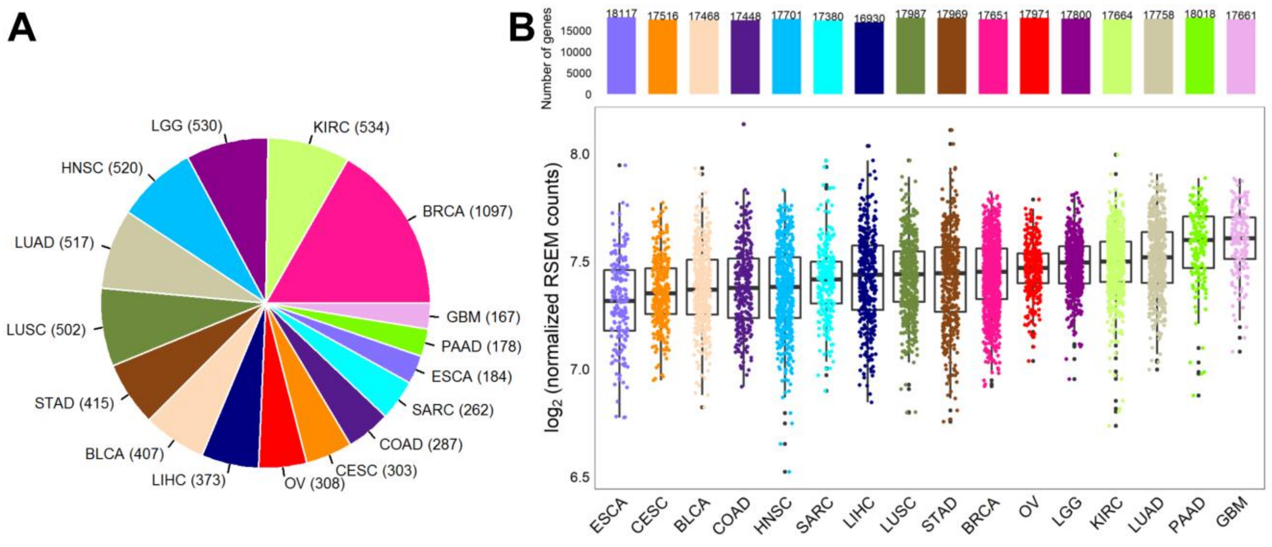

Figure S10: Overview of transcriptome expression in cancer genomes.

(A) The numbers of TCGA tumor samples in studied cancer types were shown in the pie chart. **(B)** Bar graph and boxplot show the number of detectable genes and their expression profiles in each cancer type respectively. Cancer types are ordered based on their median gene expression level.

Tables

Table S1: Comparison of co-expressed degree among protective, harmful, and non-SIGs in cancers.

BLCA	Non-SIGs	Protective	Harmful
Non-SIGs	12.30	1.20E-02	9.61E-01
Protective	-0.26	10.30	1.22E-01
Harmful	0.10	0.36	13.19

BRCA	Non-SIGs	Protective	Harmful
Non-SIGs	14.25	1.25E-05	2.53E-10
Protective	-0.28	11.74	2.86E-14
Harmful	0.53	0.80	20.51

CESC	Non-SIGs	Protective	Harmful
Non-SIGs	10.65	1.23E-01	2.47E-05
Protective	-0.23	9.10	1.66E-05
Harmful	0.26	0.49	12.79

COAD	Non-SIGs	Protective	Harmful
Non-SIGs	13.69	3.20E-03	6.67E-06
Protective	0.31	17.03	2.42E-07
Harmful	-0.46	-0.77	9.97

ESCA	Non-SIGs	Protective	Harmful
Non-SIGs	9.92	3.04E-03	6.83E-01
Protective	-0.77	5.82	1.24E-02
Harmful	0.20	0.97	11.40

GBM	Non-SIGs	Protective	Harmful
Non-SIGs	10.74	9.59E-03	1.61E-03
Protective	0.28	13.01	2.11E-05
Harmful	-0.53	-0.80	7.46

HNSC	Non-SIGs	Protective	Harmful
Non-SIGs	13.01	1.32E-01	2.35E-01
Protective	-0.18	11.51	6.01E-02
Harmful	0.37	0.54	16.79

KIRC	Non-SIGs	Protective	Harmful
Non-SIGs	13.75	1.17E-03	1.72E-04
Protective	-0.11	12.78	2.45E-07
Harmful	0.22	0.32	15.97

LGG	Non-SIGs	Protective	Harmful
Non-SIGs	13.61	5.83E-04	5.53E-12
Protective	-0.05	13.10	9.55E-03
Harmful	0.26	0.31	16.28

LIHC	Non-SIGs	Protective	Harmful
Non-SIGs	12.37	2.14E-03	2.63E-41
Protective	-0.38	9.52	1.07E-26
Harmful	0.87	1.25	22.58

LUAD	Non-SIGs	Protective	Harmful
Non-SIGs	13.33	2.34E-09	4.20E-15
Protective	-0.67	8.40	6.24E-24
Harmful	0.76	1.42	22.55

LUSC	Non-SIGs	Protective	Harmful
Non-SIGs	13.44	1.19E-01	1.03E-03
Protective	0.33	16.93	2.83E-04
Harmful	-0.71	-1.04	8.23

OV	Non-SIGs	Protective	Harmful
Non-SIGs	10.09	8.93E-03	3.73E-01
Protective	-0.24	8.55	2.98E-01
Harmful	-0.10	0.14	9.44

PAAD	Non-SIGs	Protective	Harmful
Non-SIGs	10.77	6.77E-05	5.59E-19
Protective	-0.25	9.05	3.49E-20
Harmful	0.55	0.80	15.76

SARC	Non-SIGs	Protective	Harmful
Non-SIGs	10.71	9.89E-05	1.62E-27
Protective	-0.45	7.82	8.69E-24
Harmful	0.76	1.21	18.13

STAD	Non-SIGs	Protective	Harmful
Non-SIGs	12.52	3.56E-05	1.24E-07
Protective	0.53	18.08	5.89E-12
Harmful	-0.56	-1.09	8.52

In each table, the cancer type is shown on the upper-left corner; the values on the diagonal line are mean co-expressed degree of genes in the corresponding category; the p-values derived from the Wilcoxon test are recorded on the upper-right triangle region; the lower-left triangle region scores are log ratio of co-expressed degree (SIGs to non-SIGs or harmful to protective SIGs).

Table S2: Comparison of centrality between harmful SIGs and non-SIGs in the static protein interaction network.

Cancer type	Centrality measures	Mean estimate (Harmful proteins)	Mean estimate (Other proteins)	*P*-value
**Pan-Cancer**	Deg	47.360	27.999	**1.52E-54**
	BC	1.10E-04	1.01E-04	**6.47E-44**
	CC	0.406	0.380	**7.06E-69**
**BLCA**	Deg	25.488	29.286	8.90E-01
	BC	6.35E-05	1.02E-04	9.88E-01
	CC	0.383	0.381	5.06E-01
**BRCA**	Deg	34.432	29.196	5.00E-01
	BC	7.35E-05	1.02E-04	2.59E-01
	CC	0.386	0.381	1.88E-01
**CESC**	Deg	31.019	29.237	3.22E-01
	BC	5.72E-05	1.02E-04	2.70E-01
	CC	0.392	0.381	**7.66E-03**
**COAD**	Deg	24.259	29.355	**1.01E-02**
	BC	5.38E-05	1.03E-04	1.26E-01
	CC	0.382	0.381	4.71E-01
**ESCA**	Deg	25.948	29.279	**9.88E-02**
	BC	4.40E-05	1.02E-04	1.19E-01
	CC	0.367	0.382	**6.49E-03**
**GBM**	Deg	17.127	29.372	**1.29E-04**
	BC	3.16E-05	1.02E-04	**2.29E-02**
	CC	0.362	0.382	**3.85E-07**
**HNSC**	Deg	26.953	29.263	2.73E-01
	BC	5.28E-05	1.02E-04	**4.32E-02**
	CC	0.392	0.381	**4.32E-02**
**KIRC**	Deg	22.689	29.479	**5.66E-02**
	BC	3.54E-05	1.04E-04	**2.64E-02**
	CC	0.376	0.382	**4.85E-03**
**LGG**	Deg	25.243	29.47	**2.35E-02**
	BC	4.96E-05	1.05E-04	2.70E-01
	CC	0.38	0.382	1.74E-01
**LIHC**	Deg	35.462	29.172	**1.89E-02**
	BC	6.48E-05	1.02E-04	**4.10E-02**
	CC	0.388	0.381	**1.68E-02**
**LUAD**	Deg	27.756	29.259	8.95E-01
	BC	6.49E-05	1.02E-04	9.15E-01
	CC	0.38	0.381	5.81E-01
**LUSC**	Deg	19.608	29.361	1.10E-01
	BC	4.58E-05	1.02E-04	6.32E-01
	CC	0.371	0.382	**3.09E-03**
**OV**	Deg	29.43	29.247	8.10E-01
	BC	6.16E-05	1.02E-04	6.54E-01
	CC	0.376	0.382	4.55E-01

**PAAD**	Deg	38.521	29.165	**4.01E-03**
	BC	9.44E-05	1.02E-04	**3.25E-02**
	CC	0.395	0.381	**2.57E-03**
**SARC**	Deg	54.77	28.814	**6.73E-09**
	BC	1.94E-04	1.00E-04	**1.19E-04**
	CC	0.396	0.381	**1.98E-08**
**STAD**	Deg	20.252	29.42	**3.09E-04**
	BC	3.38E-05	1.03E-04	**9.22E-05**
	CC	0.361	0.382	**5.48E-11**

[a]*P*-value in bold typeface represents statistical significance ($P < 0.05$).
[b]Abbreviations: Deg = degree; BC = betweenness centrality; CC = closeness centrality.

Table S3: Comparison of centrality between protective SIGs and non-SIGs in the static protein interaction network.

**Cancer type**	**Centrality measures**	**Mean estimate (Protective proteins)**	**Mean estimate (Other proteins)**	***P*-value**
**Pan-Cancer**	Deg	27.383	29.374	**3.98E-02**
	BC	5.06E-05	1.05E-04	**1.27E-02**
	CC	0.389	0.381	**5.64E-06**
**BLCA**	Deg	28.958	29.255	**1.74E-02**
	BC	4.64E-05	1.03E-04	1.13E-01
	CC	0.391	0.381	**7.78E-04**
**BRCA**	Deg	37.618	29.174	3.48E-01
	BC	8.45E-05	1.02E-04	**8.20E-02**
	CC	0.384	0.381	6.25E-01
**CESC**	Deg	30.408	29.234	6.64E-01
	BC	8.71E-05	1.02E-04	9.76E-01
	CC	0.387	0.381	7.65E-01
**COAD**	Deg	44.469	29.11	**1.95E-05**
	BC	1.04E-04	1.02E-04	**1.17E-04**
	CC	0.401	0.381	**2.64E-07**
**ESCA**	Deg	23.587	29.29	9.86E-01
	BC	3.68E-05	1.02E-04	6.55E-01
	CC	0.384	0.381	7.85E-01
**GBM**	Deg	37.962	29.105	**4.42E-04**
	BC	7.74E-05	1.02E-04	**7.57E-02**
	CC	0.39	0.381	**5.85E-04**
**HNSC**	Deg	25.244	29.306	4.62E-01
	BC	5.62E-05	1.02E-04	6.04E-01
	CC	0.383	0.381	5.98E-01
**KIRC**	Deg	33.533	29.176	6.90E-01
	BC	2.41E-04	9.94E-05	3.25E-01
	CC	0.385	0.381	4.38E-01
**LGG**	Deg	34.175	29.046	**4.48E-07**
	BC	8.11E-05	1.03E-04	**2.13E-04**

	CC	0.391	0.381	**4.73E-07**
**LIHC**	Deg	30.467	29.24	6.31E-01
	BC	1.11E-04	1.02E-04	8.44E-01
	CC	0.373	0.382	3.01E-01
**LUAD**	Deg	20.443	29.369	**2.46E-02**
	BC	2.84E-05	1.03E-04	**4.85E-02**
	CC	0.372	0.382	**2.18E-04**
**LUSC**	Deg	32.593	29.213	**2.72E-04**
	BC	5.25E-05	1.02E-04	**4.20E-03**
	CC	0.391	0.381	**2.07E-03**
**OV**	Deg	28.146	29.26	5.85E-01
	BC	4.38E-05	1.02E-04	2.75E-01
	CC	0.386	0.381	2.38E-01
**PAAD**	Deg	33.55	29.179	**1.52E-02**
	BC	9.48E-05	1.02E-04	**9.70E-02**
	CC	0.383	0.381	4.92E-01
**SARC**	Deg	23.507	29.33	7.76E-01
	BC	4.66E-05	1.03E-04	8.53E-01
	CC	0.387	0.381	6.76E-01
**STAD**	Deg	42.851	29.067	**6.06E-10**
	BC	9.33E-05	1.02E-04	**7.83E-09**
	CC	0.405	0.381	**5.18E-16**

[a] *P*-value in bold typeface represents statistical significance ($P < 0.05$).
[b] Abbreviations: Deg = degree; BC = betweenness centrality; CC = closeness centrality.

Table S4: List of significant cancer-relevant hallmarks in which the pan-cancer SIGs participate.

Hallmark	Harmful		Protective	
	p-value	odds ratio	*p*-value	odds ratio
E2F_TARGETS	1.16E-36	9.64	1.00E+00	0.16
G2M_CHECKPOINT	1.63E-35	9.34	9.92E-01	0.41
MYC_TARGETS_V2	1.25E-07	6.23	1.00E+00	0.00
MTORC1_SIGNALING	1.03E-17	5.38	9.83E-01	0.48
TNFA_SIGNALING_VIA_NFKB	1.79E-15	4.90	9.94E-01	0.40
MYC_TARGETS_V1	9.24E-14	4.59	9.12E-01	0.66
TGF_BETA_SIGNALING	2.36E-04	4.16	4.21E-01	1.23
INFLAMMATORY_RESPONSE	7.27E-10	3.72	1.00E+00	0.08
MITOTIC_SPINDLE	9.71E-10	3.67	8.60E-01	0.73
GLYCOLYSIS	9.71E-10	3.67	9.84E-01	0.48
P53_PATHWAY	8.58E-09	3.48	5.29E-02	1.57
EPITHELIAL_MESENCHYMAL_TRANSITION	3.32E-07	3.11	9.98E-01	0.32
UNFOLDED_PROTEIN_RESPONSE	2.57E-04	2.92	8.21E-01	0.72
REACTIVE_OXIGEN_SPECIES_PATHWAY	1.63E-02	2.91	9.45E-01	0.34
APICAL_JUNCTION	4.30E-06	2.82	9.22E-01	0.64
APOPTOSIS	4.99E-05	2.77	9.69E-01	0.50
COMPLEMENT	9.17E-06	2.75	9.93E-01	0.40
IL6_JAK_STAT3_SIGNALING	3.03E-03	2.75	9.95E-01	0.19
PI3K_AKT_MTOR_SIGNALING	1.11E-03	2.72	1.08E-01	1.62
HYPOXIA	3.37E-05	2.59	7.75E-01	0.82
INTERFERON_GAMMA_RESPONSE	3.37E-05	2.59	1.00E+00	0.23
APICAL_SURFACE	4.11E-02	2.56	9.38E-01	0.36
DNA_REPAIR	5.45E-04	2.54	4.95E-01	1.05
COAGULATION	9.33E-04	2.49	8.45E-01	0.71
IL2_STAT5_SIGNALING	2.07E-04	2.38	9.60E-01	0.56
KRAS_SIGNALING_UP	4.68E-04	2.28	9.94E-01	0.40
SPERMATOGENESIS	4.97E-03	2.21	4.34E-01	1.11
ALLOGRAFT_REJECTION	1.31E-02	1.81	9.99E-01	0.31
BILE_ACID_METABOLISM	8.98E-01	0.60	1.26E-03	2.58
FATTY_ACID_METABOLISM	9.51E-01	0.54	4.96E-04	2.43
ESTROGEN_RESPONSE_EARLY	9.41E-01	0.60	3.61E-04	2.29
UV_RESPONSE_DN	1.25E-01	1.50	6.00E-03	2.11
OXIDATIVE_PHOSPHORYLATION	1.00E+00	0.08	4.58E-03	1.95
HEME_METABOLISM	1.66E-01	1.35	8.29E-03	1.87
ADIPOGENESIS	9.75E-01	0.51	8.77E-03	1.86
PEROXISOME	5.40E-01	1.02	8.89E-02	1.69
ESTROGEN_RESPONSE_LATE	7.09E-02	1.53	9.85E-02	1.45
XENOBIOTIC_METABOLISM	1.20E-01	1.42	2.36E-01	1.26
ANDROGEN_RESPONSE	2.27E-01	1.41	4.12E-01	1.16
KRAS_SIGNALING_DN	8.66E-01	0.71	6.10E-01	0.95

HEDGEHOG_SIGNALING	6.13E-01	0.98	6.39E-01	0.93
MYOGENESIS	7.35E-01	0.85	6.83E-01	0.89
WNT_BETA_CATENIN_SIGNALING	9.55E-02	2.19	7.36E-01	0.77
CHOLESTEROL_HOMEOSTASIS	6.14E-02	2.00	8.31E-01	0.66
NOTCH_SIGNALING	1.13E-01	2.31	8.67E-01	0.50
PROTEIN_SECRETION	3.17E-01	1.29	9.35E-01	0.50
UV_RESPONSE_UP	1.23E-01	1.47	9.88E-01	0.40
ANGIOGENESIS	5.60E-02	2.61	1.00E+00	0.00
INTERFERON_ALPHA_RESPONSE	4.70E-01	1.10	1.00E+00	0.00
PANCREAS_BETA_CELLS	1.00E+00	0.00	1.00E+00	0.00

Table S5: Cancer type analyzed in the study and their abbreviations.

Cancer	Abbreviation
Urothelial Bladder Carcinoma	BLCA
Breast Invasive Carcinoma	BRCA
Cervical Squamous Cell Carcinoma and Endocervical Adenocarcinoma	CESC
Colon Adenocarcinoma	COAD
Esophageal Carcinoma	ESCA
Glioblastoma Multiforme	GBM
Head-Neck Squamous Cell Carcinoma	HNSC
Kidney Renal Clear Cell Carcinoma	KIRC
Low Grade Glioma	LGG
Liver Hepatocellular Carcinoma	LIHC
Lung Adenocarcinoma	LUAD
Lung Squamous Cell Carcinoma	LUSC
Ovarian Cancer	OV
Pancreatic Ductal Adenocarcinoma	PAAD
Sarcoma	SARC
Stomach Adenocarcinoma	STAD

Datasets

Dataset S1: List of statistically significantly associated survival influential genes in each cancer.

Dataset S2: List of significant confounding risk factors identified from univariate Cox regression (*P* < 0.05) in different cancer types.
